# Detection of Nontuberculous Mycobacterial Skin Infection by Next-Generation Sequencing: A Pilot Study

**DOI:** 10.3390/jcm15093504

**Published:** 2026-05-03

**Authors:** Jia-Wei Liu, Xiao Ma, Yue-Tong Qian, Jing-Wen Wang, Chen-Yu Zhu, Dong-Lai Ma

**Affiliations:** Department of Dermatology, State Key Laboratory of Complex Severe and Rare Diseases, Peking Union Medical College Hospital, Chinese Academy of Medical Sciences and Peking Union Medical College, Shuaifuyuan No.1, Dongcheng District, Beijing 100730, China

**Keywords:** metagenomic next-generation sequencing, cutaneous nontuberculous mycobacteria infections, diagnostic techniques

## Abstract

**Background:** Nontuberculous mycobacteria (NTM) skin infections pose significant diagnostic challenges in clinical practice, due to nonspecific clinical/histopathological features and limitations of conventional pathogenic detection methods. Metagenomic next-generation sequencing (mNGS) offers a promising approach but requires further evaluation. **Methods:** A prospective pilot study at Peking Union Medical College Hospital enrolled 20 patients with cutaneous NTM infection, confirmed by positive skin culture or mNGS. All patients underwent thorough clinical assessment, skin biopsy for histopathology and culture, and mNGS testing of skin tissue. Treatment was based on identified species and disease extent. Treatment outcomes were tracked. **Results:** Among 20 patients (median age 45.5 years), fingers were the most common site affected (*n* = 10), followed by forearms (*n* = 7), hands (*n* = 4), and face (*n* = 4). *Mycobacterium marinum* was the predominant pathogen (*n* = 12), associated with fish bone puncture, followed by *M. abscessus* (*n* = 4). mNGS demonstrated a substantially higher positivity rate than culture (95% [19/20] vs. 30% [6/20]) and delivered results faster. Histopathology revealed granulomatous inflammation in all cases. Nineteen patients presented with non-disseminated disease; one immunocompromised patient (GATA2 deficiency) had disseminated *M. abscessus* infection. Treatment success was achieved in 17 patients (85%) with tailored antibiotic regimens. Adverse drug effects occurred in seven patients. **Conclusions:** In this pilot study of cutaneous NTM infections, mNGS enabled more rapid diagnosis relative to conventional culture. Clinical presentation and exposure history correlate with specific NTM species. Integrating mNGS with clinical assessment significantly improves diagnosis and management.

## 1. Introduction

Mycobacterial infections, classified into three main groups, *Mycobacterium leprae*, the *Mycobacterium tuberculosis complex* (MTB), and nontuberculous mycobacteria (NTM), present a significant global health challenge. While tuberculosis remains a major concern, the escalating incidence of NTM infections has raised public health concerns [1]. NTMs encompass a diverse group of environmental pathogens causing skin and soft tissue infections, lymphadenitis, pulmonary infections, and disseminated infections [2]. Common species such as *M. avium complex*, *M. kansasii*, and *M. marinum* are ubiquitous in soil and water and can colonize medical equipment [3]. These infections predominantly affect young children and immunocompromised patients but can also affect healthy individuals. Treatment often requires extended courses of combination antibiotic therapy, sometimes complemented by surgical interventions.

This study focuses on the diagnostic gap in cutaneous NTM infections, which often present as sporotrichoid nodules with aquatic exposure, injection-related abscesses, or culture-negative granulomatous dermatitis and are frequently misdiagnosed due to nonspecific clinical and histopathological features. Previously, diagnosis mainly relied on histopathology and tissue culture; however, the nonspecific histopathological features and suboptimal sensitivity of culture often lead to delayed treatment [4]. Metagenomic next-generation sequencing (mNGS) heralds a transformative era in microbial diagnostics, offering an unbiased and comprehensive approach to interrogating the genetic landscape of microbial communities directly from clinical samples [5]. Unlike conventional methods, mNGS simultaneously amplifies and sequences all nucleic acids present in a specimen, enabling the detection of bacteria, viruses, parasites, and fungi, as well as host genetic material without prior assumptions or targeting specific sequences. While promising, mNGS application in cutaneous NTM diagnosis remains inadequately characterized, with current evidence limited to isolated case reports.

We therefore propose a practical clinical workflow: mNGS is used for early, rapid species identification while conventional culture is pending, to enable timely targeted therapy before culture results become available. Our study aimed to summarize the clinical characteristics and treatment outcomes of cutaneous nontuberculous mycobacterial (NTM) infections treated at our center from January 2022 to December 2024 and to evaluate the efficiency and diagnostic yield of next-generation sequencing compared with conventional tissue culture, while also analyzing their respective limitations.

## 2. Materials and Methods

### 2.1. Ethical Approval and Patient Consent

This prospective pilot study was conducted from January 2022 to December 2024, with the objective of exploring suspected NTM skin infections. The study adhered to the principles outlined in the Declaration of Helsinki and received approval from the Ethics Committee of Peking Union Medical College Hospital (Ethical No: S-K1588). All patients provided informed consent for the use of their information in this prospective pilot study.

### 2.2. Study Design

The study included individuals attending the dermatology outpatient clinic at Peking Union Medical College Hospital due to suspected NTM skin infections, regardless of the initial previous diagnosis in the other hospitals. Inclusion criteria included the following: 1. patients aged 18 years or older; 2. male or female participants; 3. patients provided informed consent; 4. diagnosis of NTM infection by skin tissue culture or by a positive mNGS result. Exclusion criteria included the following: 1. unwillingness to provide informed consent; 2. those with skin infections for which the pathogenesis had been identified and determined not to be caused by NTM; 3. negative results in both conventional culture and mNGS testing; 4. prior culture-confirmed NTM infections (new diagnosis or relapse).

### 2.3. Patients

Patients with suspected NTM skin infections were enrolled. Patient recruitment was conducted across outpatient, emergency, and inpatient settings, with relevant information documented via an electronic system. A thorough medical history was obtained, including epidemiological and demographic data, past medical history, possible triggers, clinical manifestations, laboratory test results, initial diagnosis, interventions, side effects, and outcomes. A complete physical examination was conducted, including baseline assessments of the skin infection, such as number, size, color, ulceration, and lymphadenopathy. Before skin biopsy, the lesion surface and surrounding normal skin were disinfected with 75% alcohol followed by povidone-iodine, with an adequate contact time to eliminate surface colonizing flora. The surgical skin biopsy was performed on each enrolled patient. Skin tissues were obtained from the skin lesions and sent for histopathological examination, bacterial culture, and mNGS, respectively. In patients with purulent lesions, an additional deep-lesion swab was collected after biopsy as a supplementary specimen.

### 2.4. Diagnosis and Treatment

NTM infections were defined as one or more positive culture or sequencing results indicating active infection from normally sterile sites. Disseminated infections were defined as evidence of NTM infection at two or more different organs. Treatment was initiated with a positive mNGS or skin culture result. Treatment mainly depended on the type of bacteria and the extent of lesion spread. A monotherapy or combined antibiotic strategy was adapted based on the previous treatment recommendations for skin NTM infections [6]. mNGS turnaround time referred to the interval from specimen receipt to issuance of the sequencing report, whereas culture time referred to the interval from inoculation to actionable microbiological result (visible growth with species identification for positive cultures). Clinical improvement was delineated as a reduction of ≥50% in lesion size, erythema, swelling, tenderness, or discharge. Clinical cure was defined as the complete resolution of active inflammation (including erythema, swelling, tenderness, and discharge), with or without residual scarring. Relapse was considered as the recurrence of active NTM infection at the same or adjacent site subsequent to documented cure. Patients were followed monthly to monitor treatment response and drug-related adverse effects until, based on clinical judgment, the dermatologist considered them fully recovered and no longer in need of pharmacological treatment. We defined the date of treatment completion as the date of the last dose of medication.

### 2.5. Tissue Culture

Lesion-derived skin tissue was homogenized without additional chemical decontamination step and cultured on Löwenstein–Jensen slants. Parallel incubation was conducted at 28–30 °C and 35–37 °C for up to 8 weeks with weekly observation in all samples.

### 2.6. Metagenomic Next-Generation Sequencing

DNA was extracted from biopsy-derived skin tissue samples and purulent specimens obtained from lesions clinically suspected of nontuberculous mycobacterial (NTM) infection using the Nucleic Acid Extraction Kit (BGI, Wuhan, China), according to the manufacturer’s instructions.

DNA libraries were prepared through sequential DNA fragmentation, end repair, adapter ligation, and PCR amplification (BGI, Wuhan, China). Library quality was assessed using the Agilent 2100 Bioanalyzer (Agilent Technologies, Santa Clara, CA, USA). After library pooling and DNA nanoball (DNB) generation, sequencing was performed on the MGISEQ-2000 platform (MGI, Wuhan, China).

Bioinformatic processing of mNGS data was conducted using PMseqTM-1 software (BGI, Wuhan, China). Quality control criteria included the following: (1) raw reads ≥ 20 million; (2) Q30 score ≥85%; and (3) positive internal control. Low-quality reads, duplicate reads, reads shorter than 35 bp, and adapter sequences were removed. Human host sequences were subtracted by alignment to the human reference genome (hg19) using the Burrows–Wheeler Aligner (BWA) [7]. The remaining high-quality nonhuman reads were aligned to an in-house pathogen metagenomic database (PMDB; BGI, Shenzhen, China), with additional filtering to exclude low-complexity sequences and potential contaminants [8]. For each detected organism, mapped read counts, genome coverage, relative abundance, and average sequencing depth at the species or strain level were calculated. Negative control was used to monitor potential contamination and background microbe database was used to filter putative false positive pathogens. Final reporting required successful completion of sequencing quality control and discrimination of true pathogens from background signals through comparison with negative controls. Samples exceeding three reads of NTM were labeled as positive, according to a previously validated internal laboratory threshold.

## 3. Results

### 3.1. Clinical Characteristics

A total of 20 NTM infection patients (11 females and 9 males) were enrolled, with a median age of 45.5 years (range 19–80 years). The median disease duration was 6 months (range, 2–36 months). The median treatment duration was 4.5 months; 18 of the 20 patients had associated symptoms such as pain, itching and tenderness. Trauma related to occupational or daily activities represented the most common precipitating factor. Two patients were seafood vendors. Three patients experienced piercing by fish bone. Two patients had undergone cosmetic injections by an unlicensed provider. Most patients are immunocompetent; one patient has GATA2 deficiency syndrome and is immunocompromised. Nineteen patients presented with localized or lymphomatic distributed lesions, while the patient with GATA2 deficiency syndrome suffered from disseminated *M. abscessus* infection, which was resistant to multi-antibiotic therapy. Almost all patients responded well clinically except for the patient with GATA2 deficiency syndrome. In all 20 patients, histological examinations composed of infectious granulomatous inflammation consisting of neutrophils, lymphocytes, epithelioid histiocytes, and multinucleated giant cells, indicating skin infection. Patients’ detailed information is summarized in Table 1.

### 3.2. Description of the Skin Eruptions

**Figure 1:**
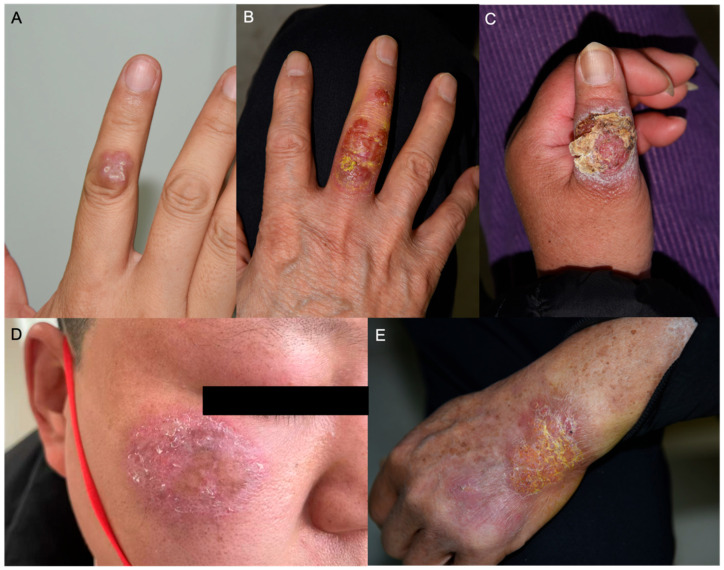
Clinical manifestations of localized nontuberculous mycobacterial skin infections. (**A**) A firm, pink nodule on the dorsal aspect of the second finger, caused by *Mycobacterium marinum*; (**B**) a moist, erythematous plaque with yellowish scale on the third finger, caused by *M. marinum*; (**C**) a large erythematous nodule with adherent yellow crust, caused by *M. marinum*; (**D**) a slightly edematous, red plaque on the cheek of a young male patient, caused by *M. fortuitum*; (**E**) a red, indurated plaque with yellow scale on the dorsal hand, caused by *M. marinum*.

**Figure 2:**
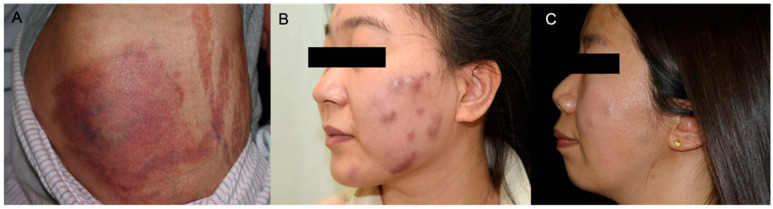
Clinical manifestations of cutaneous nontuberculous mycobacterial infections caused by *Mycobacterium abscessus*. (**A**) An extensive, erythematous, fluctuant abscess on the lower limb caused by *M. abscessus* in a patient with primary immunodeficiency; (**B**) a facial *M. abscessus* infection caused by mesotherapy in a young female, noticing dark red abscesses with some lesions coalescing into large, fluctuant nodules; (**C**) another case of facial *M. abscessus* infection after mesotherapy, showing multiple skin-colored, subcutaneous nodules.

Eleven patients presented with localized infection, manifesting as isolated brown-red nodules, with ulcerations or scales. Blood secretions and thick purulence occurred after minor trauma. Six patients had lymphocutaneous infections, presenting as multiple nodules or papules spreading along the lymphatic vessel distribution. Two patients with injection history presented a subcutaneous soft nodule with grayish pus. A patient with GATA2 deficiency syndrome and disseminated *M. abscessus* infection presented with multiple deep dermal abscesses, accompanied by local bruising and purpura. The sites of lesions predominately occurred on the upper limbs: finger and periungual (*n* = 10), forearm (*n* = 7), hand (*n* = 4) and face (*n* = 4). Typical clinical manifestations are presented in Figure 1 and Figure 2.

### 3.3. Bacterial Culture and NGS Results

While only 6 patients (30%) had a positive tissue culture, NGS showed a higher positive rate in 19 of the 20 patients (95%). mNGS generally reports within 48 h, and positive culture results typically require 7–28 days depending on species growth characteristics. Negative cultures could require prolonged incubation up to 8 weeks before final reporting. Among the 19 mNGS-positive patients, the specific species identified were *M. marinum* (*n* = 12), *M. abscessus* (*n* = 4), *M. fortuitum* (*n* = 2) and *M. ulcerans* (*n* = 1). Bacterial culture generally takes 7–28 days for the final result. Six culture-positive results were identified: three *M. marinum*, two *M. abscessus* and one *M. ulcerans*.

### 3.4. Treatment and Outcome

**Figure 3:**
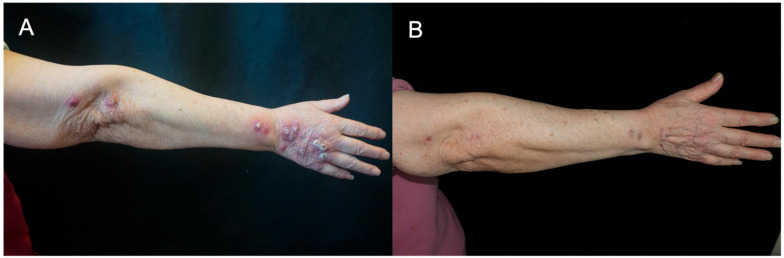
Clinical manifestations of lymphocutaneous spread of *Mycobacterium marinum* infection. (**A**) Multiple erythematous papules and nodules with overlying white crusts distributed along lymphatic pathways on the dorsum of the right hand and forearm. (**B**) Complete resolution of lesions after 12 months of combination therapy with rifampicin and clarithromycin, with residual mild hyperpigmentation.

Treatment mainly depended on the type of bacteria and the extent of lesion spread. For localized *M. marinum* skin infections, we commonly chose clarithromycin 0.5 g twice daily for at least 3 months. For those with lymphatic spread lesions, we chose Rifampin 0.15 g three times daily and clarithromycin 0.5 g twice daily (Figure 3). One patient with *M. marinum* showed drug resistance to rifampin and clarithromycin and was switched to minocycline and ethambutol. For *M. abscessus* infection, patients received multidrug regimens consisting of at least two antibiotics, primarily clarithromycin and linezolid. Complete clinical cure was achieved in 17 patients (85%), while 19 (95%) showed clinical improvement. One patient with GATA2 deficiency syndrome and *M. abscessus* infection exhibited a poor prognosis and died due to disseminated infection. Seven experienced adverse effects, including gastrointestinal irritation (*n* = 5), elevated liver enzyme (*n* = 3), dizziness (*n* = 3), and blurred vision (*n* = 2). One patient experienced thrombocytopenia, which was considered to be related to Linezolid. All adverse reactions were completely resolved after finishing the treatment.

## 4. Discussion

The incidence of cutaneous mycobacterial infections has risen globally in recent years, driven by increasing human immunodeficiency virus prevalence, widespread immunosuppressant use, population aging, and enhanced diagnostic capabilities [9]. The genus *Mycobacterium* consists of aerobic, non-motile, non-spore-forming, acid-fast bacilli with Gram-positive cell wall characteristics and is the only member of the *Mycobacteriaceae* family within the *Actinobacteria* phylum [10]. Initially classified based on growth rate and pigment production, mycobacteria can now be identified through genomic analysis, which has revealed the existence of over 170 species [11].

Mycobacterial skin infections can be attributed to various species, including MTB, *M. leprae*, and NTM. NTM are further categorized as rapidly growing (RGM) or slowly growing mycobacteria (SGM) based on colony formation speed. Recent epidemiological studies confirm the skin as the second most frequent site of NTM infection in both immunocompetent and immunocompromised individuals, preceded only by pulmonary disease in adults and lymphadenopathy in children [12,13]. Recent epidemiological studies have shown an increasing incidence of cutaneous NTM infections globally. The surge in RGM infection outbreaks has been linked to invasive cosmetic procedures like tattoos, acupuncture, pedicures, liposuction, and injections. RGM are notably resistant to standard disinfectants such as ethyl alcohol and chlorhexidine, leading to infections that can result from inadequately sterilized instruments [9,11].

Clinical manifestations and incubation periods of cutaneous NTM infections exhibit considerable heterogeneity, influenced by the mode of acquisition, bacterial load, pathogen virulence, and host immune status. The clinical symptoms exhibit remarkable variability and are contingent upon the type (species and strain) and quantity of pathogens (multibacillary vs. paucibacillary forms), along with the patient’s immune competence [10]. Nodules and plaques represent the predominant morphological features observed in cutaneous NTM infections. Variations in presentation encompass abscesses, papules, patches, cellulitis-like lesions, and sporotrichoid spread. In our cohort, sporotrichoid or lymphocutaneous spread on the upper extremities after aquatic exposure or fish-spine trauma was most commonly associated with Mycobacterium marinum. Periungual or finger nodules with chronic indolent inflammation, especially in patients handling seafood or aquariums, were also frequently linked to Mycobacterium marinum. In contrast, fluctuant facial or soft-tissue abscesses after cosmetic injections or mesotherapy were mainly associated with Mycobacterium abscessus in our series. Notably, the patient with a negative mNGS result presented with a solitary nodule on the index finger, with histopathological examination accorded with granulomatous inflammation, and culture identified infection with the Mycobacterium marinum. Main clinical differential diagnosis includes cutaneous tuberculosis, deep fungal infection, sporotrichosis, and leprosy. In our study, fingers were the most commonly affected site, followed by arms, hands, and faces. *M. marinum* was the most frequent species, followed by *M. abscessus*, *M. fortuitum* and *M. ulcerans*. Most patients with *M. marinum* reported a definite history of puncture by seafood spines or marine organisms, while two patients with *M. abscessus* had undergone cosmetic injections administered by unauthorized practitioners. One patient with *M. abscessus* had an inherited immune deficiency due to GATA2 deficiency.

Histopathological examination of NTM infection typically presented with nonspecific granulomatous inflammatory cell infiltrate, including neutrophils, lymphocytes, plasma cells, and histiocytes, occasionally with caseous necrosis. Histopathologic diagnosis is often combined with Ziehl–Neelsen staining to identify bacterial presence, although this method does not specify the bacterial species. Histopathological findings show correlation with the immune status of patients. Immunocompetent patients tend to present with more well-demarcated granulomas and increased plasma cell infiltration. Meanwhile, although histological presentation showed overlap between MTB and NTM infections. Neutrophil infiltration, suppurative granuloma, small vessel proliferation, and increased numbers of bacilli were found to be associated with nontuberculous mycobacterial infections, while giant cells, plasma cells, tuberculoid granulomas, and necrosis were associated with tuberculosis [10]. In our study, patients’ histological findings were consistent with previously reported NTM infections, demonstrating mixed inflammatory cell infiltration with or without typical granulomatous patterns.

Diagnosing cutaneous NTM infections remains challenging due to the absence of distinct species-specific clinical and histopathological features, as well as the low positivity rate in pathogen detection. Consequently, diagnosis relies heavily on etiological examination. Traditional detection methods include histopathology, skin tissue culture, and PCR-based techniques. While tissue culture represents the gold standard for species identification and drug susceptibility testing, its utility is constrained by prolonged turnaround times (weeks) and variable unsatisfactory positivity rates (33–85% across studies) [14]. In our study, to maximize tissue culture positivity rates, we performed a surgical biopsy, and the specimen was immediately processed, supplemented by dual-temperature incubation (28–30 °C and 35–37 °C) to prevent detection gaps. Despite these measures, culture positivity remained low at 30% (6/20 cases), underscoring the persistent limitations of conventional methods. Recent advancements involve using multiplex amplifications and DNA microarray chip technology for rapid detection. DNA microarray chip technology has emerged as a promising tool for the rapid detection of mycobacterial species, enabling high throughput and rapid detection of mycobacterial isolates in clinical specimens [9,15]. DNA microarray chip assays provide significantly higher sensitivity (91.67%) compared to traditional skin tissue culture methods (70%) while maintaining 100% specificity in cutaneous mycobacterial infection [9].

Non-targeted identification of microbes is now possible based on NGS techniques, which allows deep sequencing of biological samples and identification of pathogens without a priori knowledge. This agnostic sequencing strategy not only enhances diagnostic sensitivity and specificity but also holds promise for uncovering novel pathogens and informing personalized therapeutic interventions. Metagenomic next-generation sequencing (mNGS) is revolutionizing clinical microbiology by providing a comprehensive approach to pathogen detection and characterization directly from clinical samples. In a recent study, mNGS successfully detected NTM in 27 patients, which conventional culture methods yielded a negative result in 22 patients [16]. Although mNGS offers a rapid and highly accurate diagnostic approach, several challenges persist. False-negative results in metagenomic next-generation sequencing (mNGS) for pathogen detection represent a common problem. First of all, extracting large quantities of intact, pure genomic DNA is challenging with NTM due to their hardy, lipid-laden mycobacterial cell wall [17]. Then, the overwhelming presence of human host DNA/RNA is also a significant factor contributing to false-negative results. Typically, >99% of sequenced reads are derived from the human host, thus limiting the overall analytical sensitivity for pathogen detection due to the relative scarcity of microbial nonhuman reads. Additionally, low pathogen nucleic acid concentrations in clinical samples can result in insufficient coverage for detection, as the sensitivity of mNGS depends on the assay’s ability to efficiently extract and prepare libraries from genomic material present in samples. Interfering substances commonly found in clinical specimens, such as heme, proteins at high levels, and bilirubin, can inhibit nucleic acid extraction or enzymatic activities, leading to false-negative results. Furthermore, when certain organisms appear as part of the background contamination in mNGS assays, distinguishing truly positive samples from background noise may require higher organism concentrations to be present, potentially causing low-level infections to be missed. These technical limitations may all lead to false-negative results and delayed diagnosis in clinical practice [18]. In this pilot cohort, mNGS showed a substantially higher positive rate (95% vs. 30%) and faster turnaround compared with conventional skin tissue culture.

The management of NTM skin infections lacks standardized treatment protocols, although multidrug regimens are commonly recommended. For Mycobacterium avium complex (MAC) and *M. abscessus*, the use of three active agents, identified via in vitro susceptibility testing, is advised. Treatment durations for skin infections are generally shorter than those for pulmonary NTM: 6 to 12 months for MAC and *M. abscessus*, a minimum of 4 months for *M. chelonae* and *M. fortuitum*, and 3 to 4 months for *M. marinum* [19].

The management of *M. abscessus* presents significant clinical challenges due to its diverse virulence factors and intrinsic resistance mechanisms. While *M. abscessus* is inherently resistant to most antituberculous agents, it frequently exhibits susceptibility to clarithromycin, amikacin, and cefoxitin. Case reports have documented the successful use of bedaquiline, omadacycline, tedizolid, and linezolid in the treatment of cutaneous NTM infections [20].

Surgical interventions, including excision and drainage, serve as either a curative treatment or adjunctive therapy combined with antimicrobial chemotherapy. For *M. marinum* infections, surgical intervention should be considered when deeper tissue involvement occurs or when medical therapy demonstrates inadequate therapeutic response [21]. For *M. abscessus* infections, surgical debridement of cutaneous or subcutaneous lesions is generally recommended due to the organism’s propensity for biofilm formation and multidrug resistance [13,22]. However, in our study, patients with *M. abscessus* infection following facial cosmetic procedures were particularly reluctant to undergo surgical interventions, including drainage or biopsy, likely due to concerns about further compromising facial esthetics.

Despite advances in molecular diagnostics, the potential for false-negative results persists with mNGS. Patients presenting with consistent clinical symptoms or biopsy findings, yet yielding negative microbiological test results and tissue culture, present ongoing diagnostic challenges. The standardization of methods and interpretation of results require interdisciplinary collaboration among clinical microbiology, computational biology, infectious diseases, and other relevant fields [23].

As modern medicine increasingly embraces AI-supported diagnostic tools, the implications of our pilot study extend beyond validating the potential clinical value of mNGS and point to a broader future where artificial intelligence can integrate complex clinical, histopathologic, and molecular data to refine the diagnosis of cutaneous infections. In routine clinical practice, physicians and residents already use image-recognizing large language models as informal adjuncts for evaluating inflammatory and infectious skin lesions. Recent studies by Boostani et al. [24,25] have demonstrated robust performance of such AI systems in skin image interpretation, supporting their potential to assist dermatological diagnosis. Image-recognizing artificial intelligence could help standardize recognition of key clinical patterns associated with NTM infection, including nodules, plaques, abscesses, and lymphocutaneous spread. Multimodal AI systems may eventually integrate lesion images, exposure history, histopathological findings, and mNGS sequencing outputs to enable earlier and more accurate species-directed diagnosis. Importantly, AI would not replace microbiologic confirmation; instead, it could augment the diagnostic pathway by helping clinicians promptly suspect NTM infections and prioritize advanced testing such as mNGS. This approach directly supports our study’s conclusion that integrating mNGS with careful clinical assessment significantly improves the diagnosis and management of cutaneous NTM infections.

The integration of precision medicine diagnostics, combining pathogen genomes, host transcriptomes, and microbiome data, promises personalized treatment approaches. However, widespread adoption relies on addressing challenges such as cost-effectiveness, technical standardization, and the need for specialized expertise in the interpretation of complex molecular data. Future multi-center, prospective diagnostic accuracy studies enrolling unselected consecutive patients with suspected cutaneous NTM infection are needed to validate the true diagnostic performance and clinical utility of mNGS.

## 5. Limitation

The limitation of this study is that, despite mNGS providing a sufficient method for pathogen detection and confirmation of cutaneous NTM infections, patients presenting with consistent clinical symptoms or biopsy findings, yet yielding negative microbiological test results and tissue culture, were excluded according to our criteria. This exclusion may have led to an incomplete or potentially biased representation of the disease. In the meantime, because mNGS positivity constituted one enrollment pathway, the observed positivity rate may overestimate real-world diagnostic performance. The study design precludes true diagnostic accuracy claims and may overestimate mNGS performance. Furthermore, we did not compare mNGS with other molecular methods for NTM diagnosis (such as PCR targeting hsp65 or atpE) or with Ziehl–Neelsen or fluorescent staining. Also, the present study did not include antibiotic susceptibility testing, which failed to correlate the microbial species with resistance profiles and treatment outcomes. Other limitations of this study include its single-center, pilot design and small sample size, which may limit the generalizability of the findings. In addition, the high cost of mNGS may restrict its widespread clinical application in resource-limited settings. A larger prospective diagnostic accuracy study enrolling all clinically suspected patients regardless of final microbiological results is warranted to determine formal sensitivity, specificity, and predictive values.

## 6. Conclusions

This single-center, prospective pilot study of 20 patients with culture- or mNGS-confirmed cutaneous NTM infection demonstrates that metagenomic next-generation sequencing (mNGS) achieves a markedly higher diagnostic positivity rate (95% [19/20]) and faster turnaround time compared with conventional skin tissue culture in the detection of cutaneous NTM pathogens. Our findings support that integrating mNGS with clinical, exposure, and histopathologic assessment enables earlier, more accurate species identification and targeted management of cutaneous NTM infections.

## Figures and Tables

**Table 1:** Clinical information of enrolled patients.

Case No.	Gender	Age	Initial Diagnosis	Underlying Disease	Location	Time from Onset to Consultation (Months)	Predisposing Factor	Culture Result	Sequencing Result	Treatment	Treatment Duration (Months)	Treatment Outcome
1	M	24	Periungual abscess	—	Periungual	2	Bone fracture	Negative	*M. fortuitum*	Minocycline	2	Cured
2	F	72	Sporotrichosis	—	Dorsum of hand and forearm	3	Trauma	Negative	*M. marinum*	CLR + RIP (2 months, poor effect), then minocycline + ethambutol + CLR	6	Cured
3	M	80	Sporotrichosis	—	Dorsum of hand	36	Plant thorn injury	Negative	*M. marinum*	CLR	4	Cured
4	F	30	Sporotrichosis	—	Face	24	—	Negative	*M. abscessus*	CLR + LZD	4	Partial improvement
5	M	72	Sporotrichosis	Diabetes mellitus	Finger	12	Trauma	Negative	*M. marinum*	CLR	5	Cured
6	M	19	NTM infection	GATA2 deficiency syndrome	Buttocks, abdomen, bone	12	Reptile keeper	*M. abscessus*	*M. abscessus*	Imipenem-cilastatin + amikacin + LZD	11	Ineffective, died of disseminated infection
7	F	45	Sporotrichosis	—	Finger, dorsum of hand, forearm	6	Trauma	Negative	*M. marinum*	CLR + RIP	6	Cured
8	M	46	Furuncle	—	Finger	6	Mosquito bite	*M. marinum*	*M. marinum*	CLR	4	Cured
9	M	32	Swimming pool granuloma	—	Finger	36	Seafood vendor	*M. marinum*	Negative	CLR	3	Cured
10	M	26	Systemic lupus erythematosus	—	Face	12	Trauma	Negative	*M. fortuitum*	CLR	3	Cured
11	F	25	NTM infection	—	Face	4	Mesotherapy	*M. abscessus*	*M. abscessus*	CLR + LZD	12	Cured
12	F	33	NTM infection	—	Face	2	Mesotherapy	Negative	*M. abscessus*	CLR + LZD	6	Partial improvement
13	F	57	Infectious granuloma	—	Forearm, finger	8	Seafood vendor	*M. marinum*	*M. marinum*	CLR + RIP	4	Cured
14	F	79	NTM infection	—	Forearm, finger	3	—	Negative	*M. marinum*	CLR + RIP	12	Cured
15	M	40	NTM infection	—	Forearm, finger	3	Fish bone puncture	Negative	*M. marinum*	CLR + RIP	3	Cured
16	F	51	Sporotrichosis	—	Forearm	3	—	Negative	*M. marinum*	CLR	3	Cured
17	M	31	Infectious granuloma	—	Dorsum of hand	6	Tropical fish and tortoise keeper	*M. ulcerans*	*M. ulcerans*	Minocycline + ethambutol + CLR	24	Cured
18	F	53	Lipoma	—	Forearm	12	—	Negative	*M. marinum*	Surgical excision + CLR + RIP	6	Cured
19	F	55	Fungal infection	—	Finger	24	Fish bone puncture	Negative	*M. marinum*	CLR + RIP	6	Cured
20	F	56	Skin infection	—	Finger	3	Fish bone puncture	Negative	*M. marinum*	CLR	3	Cured

NTM, nontuberculous mycobacteria; RIP, Rifampin; CLR, Clarithromycin; LZD, Linezolid; GATA2, GATA binding protein 2.

## Data Availability

The data that support the findings of this study are available on request from the corresponding author. The data are not publicly available due to privacy or ethical restrictions.
